# High expression of TRIM24 predicts worse prognosis and promotes proliferation and metastasis of epithelial ovarian cancer

**DOI:** 10.1186/s13048-022-00948-8

**Published:** 2022-02-01

**Authors:** Liwei Zhang, Hong Chen, Baijuan Ding, Wei Jiang

**Affiliations:** 1 Department of Gynecology, Obstetrics and Gynecology Hospital of Fudan University, Shanghai, 200011 China; 2 Shanghai Key Laboratory of Female Reproductive Endocrine Related Diseases, Shanghai, 200011 China; 3 The People’s Hospital of Danyang, Affiliated Danyang Hospital of Nantong University, Danyang, Jiangsu Province, 212300 China; 4 Department of Obstetrics and Gynecology, Tengzhou Central People’s Hospital, Tengzhou, Shandong Province, 277599 China

**Keywords:** Epithelial ovarian cancer (EOC), TRIM24, Proliferation, Metastasis, Prognosis

## Abstract

**Background:**

Tripartite Motif-Containing 24 (TRIM24) is a member of the tripartite motif family. TRIM24 is claimed aberrantly activated in a number of cancers, such as breast cancer, prostate cancer and lung cancer. However, the expression of TRIM24 in epithelial ovarian cancer (EOC) and its relationship with prognosis remain unclear. In this study, we investigated the expression pattern and underlying clinical significance of TRIM24 in EOC.

**Results:**

Data from Oncomine and immunohistochemistry of tissue samples demonstrated that TRIM24 expression was obviously elevated in ovarian carcinoma compared with normal ovary tissues. Elevated TRIM24 expression was closely correlated with serum CA-125 (*P* = 0.0294), metastasis (*P* = 0.0022), FIGO (International Federation of Gynecology and Obstetrics) stage (*P* = 0.0068) and Ki-67 level (*P* = 0.0395). Kaplan–Meier survival analysis found that TRIM24 expression increased inversely with the clinical prognosis of patients with EOC. Moreover, colony formation and CCK-8 assays showed that TRIM24 promoted EOC cell growth, and tumorigenic experiments in nude mice showed that TRIM24 knockdown inhibited tumor growth in vivo. The Spearman’s correlations revealed that the expression of TRIM24 was significantly correlated with levels of Ki-67 (*P* = 0.01), at a correlation coefficient of 0.517. Wound-healing and transwell migration assays demonstrated TRIM24 facilitated cell migration. Mechanism studies showed that TRIM24 could promote the phosphorylation level of Akt and the process of EMT.

**Conclusion:**

Our results confirmed that TRIM24 could predict poor prognosis of EOC patients and promote tumor progression by regulating Akt pathway and EMT. TRIM24 may be used as a new prognostic marker for EOC and may provide a new strategy for targeted therapy of epithelial ovarian cancer.

## Background

Ovarian cancer is the second most common cause of death in women with gynecological cancer worldwide. Epithelial ovarian cancer (EOC) accounts for 90% of the total ovarian cancer [1]. Due to lack of effective early screening methods and specific warning symptoms, most patients were diagnosed as phase FIGO III (51%) or FIGO IV (29%), the corresponding 5-year cause specific survival rates were 42% and 26%, respectively [2]. Cytoreductive surgery and platinum taxane combined chemotherapy are the mainstream treatment methods. A majority of patients who presented advanced disease would have drug resistance and recurrence within 18 months, eventually leading to death [3]. Further research on molecular and cellular profiling is helpful to identify new biomarkers for the diagnosis and treatment of EOC.

Tripartite Motif-Containing 24 (TRIM24) has characteristic of the TRIM family of proteins, amino-terminal RBCC domains (Ring, B-Box, Coiled-Coil), and a TIF1 sub-family-defining PHD-bromodomain, it is an E3 ubiquitin ligase as well as a transcription co-regulator [4, 5]. Its N-terminal TRIM motif includes an important zinc-binding domain-the RING region, which is involved in ubiquitylation and degradation of the major transcription factor p53 [6, 7]. TRIM24 carrying a tandem plant homeodomain finger–bromodomain (PHD-BROMO) at its C-terminus, through which it is able to recognize the specific modification pattern, namely histone 3 unmethylated at K4 (H3K4me0) and acetylated at K23 (H3K23ac) as a reader protein of chromatin-associated epigenetic [8]. Through its conserved LxxLL motif TRIM24 can mediate transcriptional control by interacting with the activation function 2 (AF-2) regions of several nuclear receptors, such as the estrogen, retinoic acid, vitamin D3 receptors and progesterone receptors [9–11].

TRIM24 is claimed aberrantly activated in a variety of cancer lineages and over-expressed TRIM24 is associated with tumorigenesis and disease progression [8, 12, 13]. TRIM24 promotes prostate cancer proliferation at low androgen conditions and bolsters androgen receptor signaling. Moreover, androgen receptor and TRIM24 co-activated genes are significantly upregulated in castration-resistant state prostate cancer [14]. Recent research unveiled that TRIM24 upregulated in glioblastoma, and it accelerated tumor growth and induced chemotherapy resistance via the phosphatidylinositide 3-kinase (PI3K)/Akt pathway [15]. However, the expression of TRIM24 in EOC and its effect on the proliferation and metastasis of EOC cells and the underlying mechanism remain unclear.

In this study, we investigated the expression pattern of TRIM24 in EOC by immunohistochemistry and analyzed the relationship of TRIM24 expression with clinicopathological parameters. Moreover, we knocked down TRIM24 in SKOV3 cells and overexpressed TRIM24 in CaOV-3 cells to explore the affection of TRIM24 on the proliferation and metastasis capacity of EOC cells. This study clarified the role of TRIM24 in EOC, and provides a theoretical basis for the application of TRIM24 in the diagnosis and treatment of EOC.

## Results

### Expression of TRIM24 in epithelial ovarian cancer tissues

**Fig. 1:**
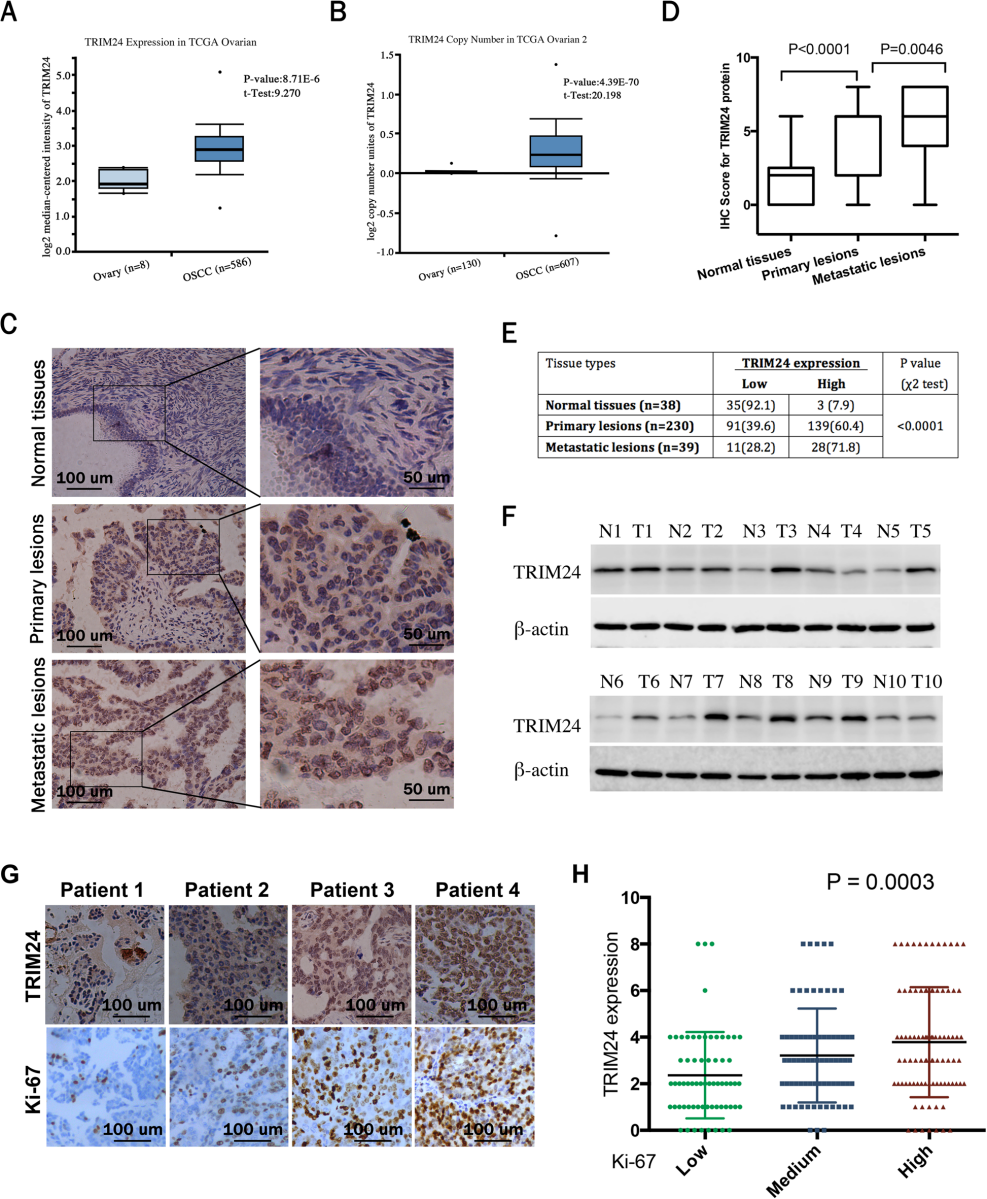
Expression of TRIM24 in EOC tissues. **a** TRIM24 expression in TCGA Ovarian database from Oncomine, *P* = 8.71E-6, t = 9.27. **b** TRIM24 expression in TCGA Ovarian 2 database derived from Oncomine, *P* = 4.39E-70, t = 20.198. **c** IHC analysis of TRIM24 protein level in normal ovarian tissues, EOC primary cancer tissues and metastatic tissues. Representative images are shown at 200 × , 400 × magnification, respectively. **d** The box plots of IHC score of TRIM24 protein in three groups of normal ovarian tissues, EOC primary cancer tissues, and  metastatic tissues. **e** The high-expression rate of TRIM24 protein in normal ovarian tissues, EOC primary cancer tissues and metastatic tissues. **f** Western blot analysis of the TRIM24 level in 10 selected pairs EOC tissues and corresponding normal ovarian tissues. **g** IHC analysis of TRIM24 and Ki-67 protein level in corresponding EOC tissues. Representative images are shown at 200 × magnification. **h** The scatter diagram of IHC score of TRIM24 protein in Ki-67 low, medium and high expression groups, *P* = 0.0003

Firstly, we analyzed TCGA Ovarian and TCGA Ovarian 2 database derived from Oncomine to investigate the expression pattern of TRIM24 in EOC. As shown in Fig. 1a and 1b, compared to normal ovary tissues, TRIM24 mRNA expression had a significant enhancement in ovarian serous cystadenocarcinoma (TCGA Ovarian database: *P* = 8.71E-6, t = 9.27, and TCGA Ovarian 2 database: *P* = 4.39E-70, t = 20.198). Secondly, we employed IHC to explore the expression of TRIM24 protein in TMAs, which included 38 cases of normal ovarian tissue, 230 cases of EOC primary lesions, and 39 cases of metastatic lesions. The results illuminated that TRIM24 mainly located in the nucleus of EOC cells (Fig. 1c). The Median IHC score of TRIM24 were gradually increased in the three groups of normal ovarian tissues, EOC primary cancer tissues and metastatic tissues (Fig. 1d). The metastatic lesions had the markedly highest high-expression rate of TRIM24 protein (71.8%), which was much higher than that of primary EOC and normal tissues (*P* < 0.0001) (Fig. 1e). Western blot test of samples from 10 patients with FIGO IA stage EOC showed that the expression level of TRIM24 in EOC tissues was significantly higher than that in corresponding normal tissues (Fig. 1f). These results suggested that TRIM24 might play an oncogene role in the tumorigenesis and development of EOC.

### Correlation between TRIM24 expression and EOC clinicopathological parameters

To explore the clinical significance of TRIM24 expression in EOC, the associations between clinicopathological features and TRIM24 expression were summarized in Table 1. TRIM24 expression had a strong correlation with the adverse clinicopathological features of EOC, including serum CA-125 (*P* = 0.0294), metastasis (*P* = 0.0022), FIGO stage (*P* = 0.0068) and Ki-67 level (*P* = 0.0395). The further analysis of Spearman’s correlations revealed that the expression of TRIM24 was significantly correlated with levels of Ki-67 (*P* = 0.01), at a correlation coefficient of 0.517. Ki-67 levels were positively correlated with TRIM24 expression in EOC patients. We further analyzed the relationship of expression level between TRIM24 and Ki67. In Fig. 1g, we can see that the immunohistochemical intensity of TRIM24 and Ki67 increased gradually, and there was a positive correlation between TRIM24 and Ki67. According to Ki67 IHC score, the samples were divided into three groups. With the enhancement of Ki-67 IHC score, TRIM24 IHC score was gradually strengthened (Fig. 1h). There was significant difference among the three groups (*P* = 0.0003). The data suggested that TRIM24 might promote EOC cell proliferation and metastasis.

**Table 1:**
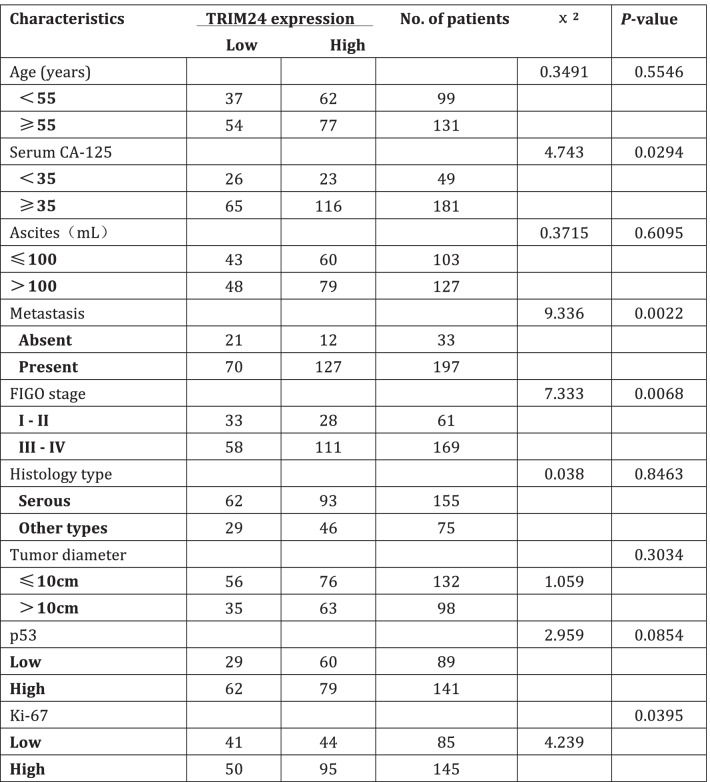
Relationship between TRIM24 protein level and patient’s clinicopathologic characteristics

### Relationship between TRIM24 expression and prognosis in EOC patients

**Fig. 2:**
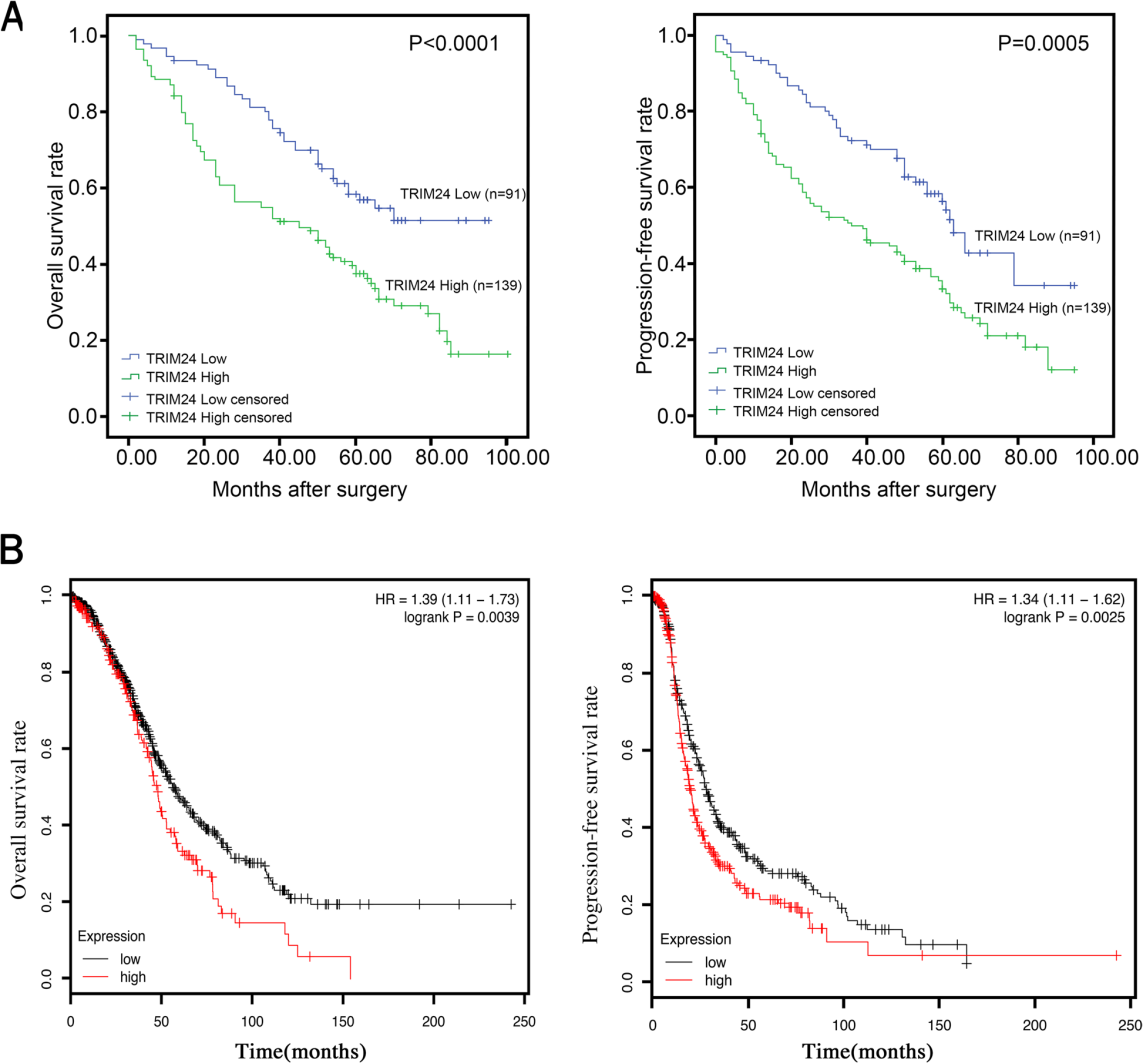
Relationship between TRIM24 expression and prognosis in patients with EOC. **a** Kaplan–Meier survival curves showed that TRIM24 expression was dramatically associated with EOC patients’ OS (*P* < 0.0001) and PFS (*P* = 0.005). P-values were calculated by log-rank test. **b** The impact of TRIM24 on the prognosis of EOC patients according to data from http://kmplot.com

The relations between TRIM24 expression and overall survival (OS) and progression-free survival (PFS) were analyzed via Kaplan–Meier method. At the end of follow-up, 132 patients were deceased, and the median observation time was 50.0 months (2–100 months). There were 91 patients with low TRIM24 expression and 139 patients with high TRIM24 expression. The percentages of deaths in low and high TRIM24 expression groups were 42.9% and 66.9%, and the mean survival time of the dead patients in the two groups were 36.0 and 29.6 months, respectively. As shown in Fig. 2a, Kaplan–Meier test verified that TRIM24 expression was dramatically associated with EOC patients’ OS (*P* < 0.0001)  and PFS (*P* = 0.005). Furthermore, we analyzed the impact of TRIM24 on the prognosis of EOC patients according to data from web of http://kmplot.com. As shown in Fig. 2b, the OS rate and PFS rate of patients with high TRIM24 expression was significantly lower than that of patients with low TRIM24 expression, with a hazard ratio of 1.39 (1.11–1.73), *P* = 0.0039 and 1.34 (1.11–1.62), *P* = 0.0025, respectively. The mean overall survival time of TRIM24 high expression group was 47.94 months, while that of TRIM24 low expression group was 56.17 months; the progression free survival time was 19.43 months in the TRIM24 high expression group and 27.98 months in the TRIM24 low expression group. In sum, the data demonstrated that high TRIM24 expression might be an unfavorable predictor for prognosis in EOC patients.

### TRIM24 promotes EOC cell proliferation in vitro

**Fig. 3:**
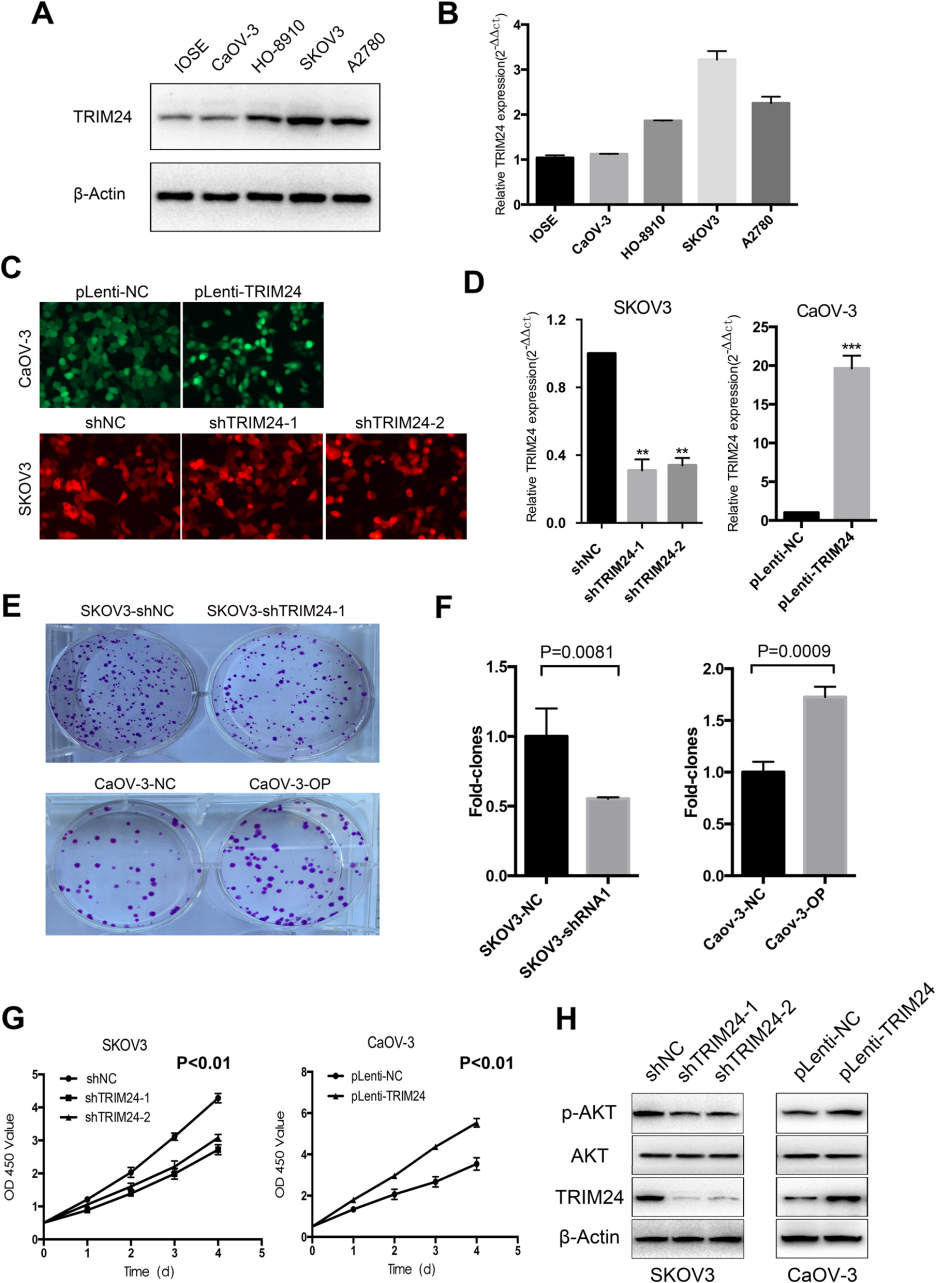
TRIM24 promotes EOC cell proliferation in vitro. **a** and** b** Western blotting and qRT-PCR analysis of TRIM24 protein and mRNA expression in IOSE and a panel of EOC cell lines. **c** The transfection efficiency of SKOV3 and CaOV-3 cells was detected by fluorescence imaging (magnification, × 200). **d** qRT-PCR analysis of TRIM24 knockdown in SKOV3 and overexpression in CaOV-3 cells. **e** Representative images of in vitro colony formation assay of SKOV3 and CaOV-3 cells. **f** Quantification of the EOC colonies shown in **e** respectively. **g** The effect of TRIM24 on EOC cell proliferation was determined by CCK-8 assay. **h** Western blot showed that knockdown of TRIM24 in SKOV3 cells could inhibit the phosphorylation of Akt, while overexpression of TRIM24 in CaOV-3 cells could promote the phosphorylation of Akt

In order to explore the effect of TRIM24 on the biological behavior of EOC cells, we examined the expression level of TRIM24 protein and mRNA by Western blot and qRT-PCR in ovarian immortal cell line IOSE and a panel of EOC cell lines (Fig. 3a and b). TRIM24 expression in EOC cell lines was significantly higher than that in IOSE cells. This result suggests that TRIM24 maybe a tumor-promoting gene in EOC. Further, we conducted lentivirus-mediated knockdown expression of TRIM24 in SKOV3 and overexpression of TRIM24 in CaOV-3 cells, respectively. As shown in Fig. 3c, the fluorescence intensity revealed that the efficiency of plasmid transfection was more than 90%. The qRT-PCR results demonstrated that compared with the control group, the TRIM24 mRNA level was reduced by 69% and 66% in SKOV3 cells and increased by 19.63 times in CaOV-3 cells (Fig. 3d). Moreover, Western blotting showed that TRIM24 protein expression decreased in SKOV3 cell line and increased significantly in CaOV-3 cell line (Fig. 3h). These results demonstrated that TRIM24 knockdown and overexpression cell models were successfully constructed. The colony formation results demonstrated that silencing TRIM24 led to decreased colony formation ability of SKOV3 cells, conversely, enforced expression of TRIM24 notably promote colony formation ability of CaOV-3 cells (Fig. 3e and f). The CCK-8 assay showed that TRIM24 could obviously promote the proliferation of EOC cells (Fig. 3g). Recent studies on other tumors have shown that TRIM24 may promote tumorigenesis by affecting Akt pathway. Therefore, we explored in EOC and the results showed that interfering the expression of TRIM24 would inhibit the phosphorylation of Akt, while overexpression of TRIM24 could increase the phosphorylation level of Akt. Taken together, these results indicated that TRIM24 could promote the proliferation of EOC cells by affecting Akt pathway in vitro.

### Overexpression of TRIM24 promotes proliferation of EOC cell in vivo

**Fig. 4:**
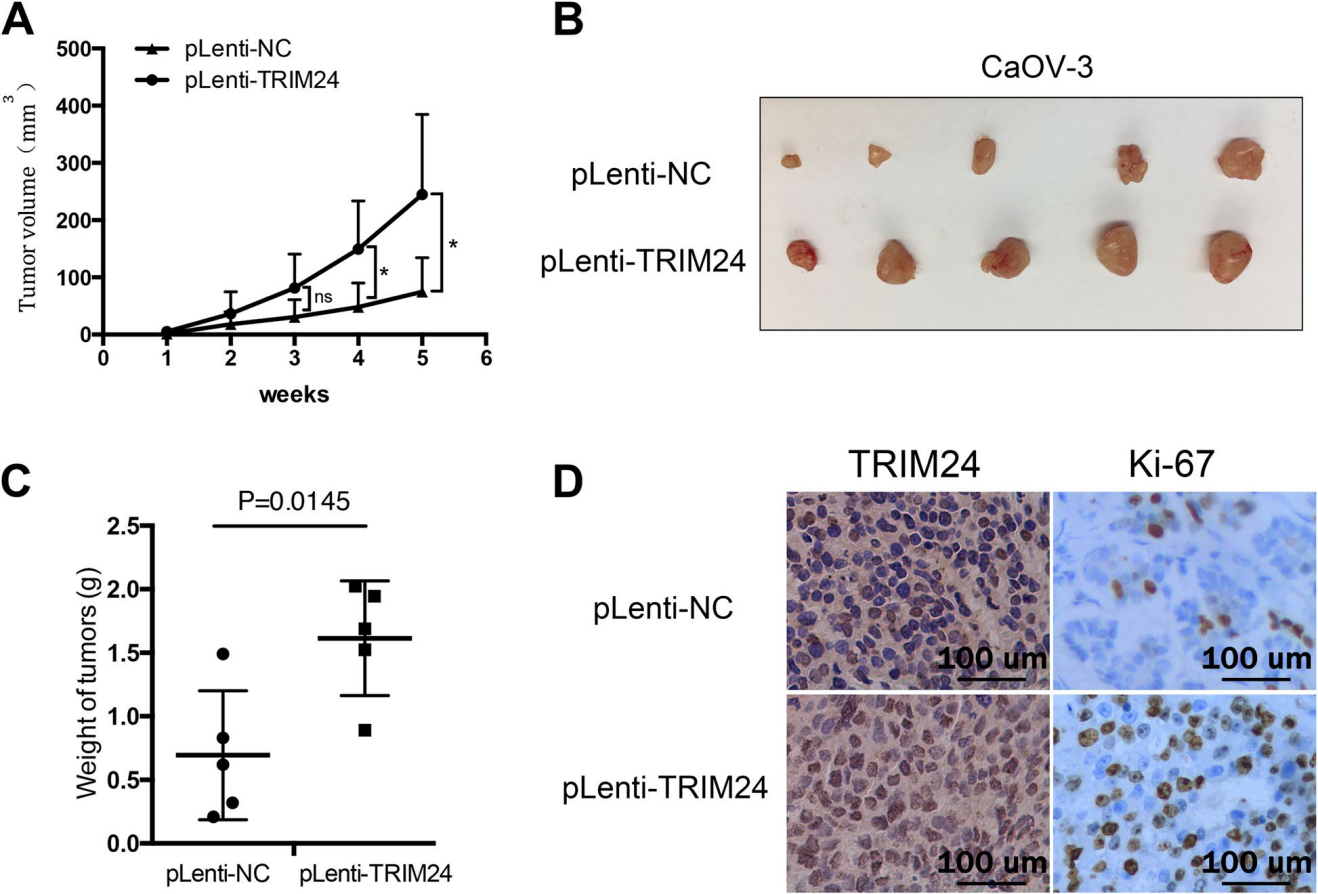
Overexpression of TRIM24 promotes proliferation of EOC cell in vivo. **a** Overexpression of TRIM24 promotes the tumorigenicity of CaOV-3 cells in the xenograft mouse model (*P* < 0.05). **b** and **c** The weights of the tumor nodules from the TRIM24 overexpressed CaOV-3 cells are significantly heavier than that of the control CaOV-3 cells. **d** Representative images of IHC staining of TRIM24 and Ki-67 from tumor nodules formed by mice inoculated with control CaOV-3 cells or TRIM24 overexpressed CaOV-3 cells (*P < 0.05, ns: no sense)

To further validate the results of cell growth tests in vitro, tumorigenicity tests were carried out in nude mice using TRIM24 overexpressed and control CaOV-3 cells. One week after subcutaneous inoculation of tumor cells, tumor nodules could be seen. During the next second, third, fourth and fifth weeks, the tumor nodules meridians were measured and recorded. The results showed that the tumor volume of TRIM24 overexpression group was significantly larger than that of control group. The average tumor volume of the TRIM24 overexpression group was nearly three times than that of the control group, namely 233.1 mm^3^ versus 86.5 mm^3^ (*P* < 0.05) (Fig. 4a). At the fifth week, we sacrificed the mice, collected the tumor nodules and weighed them. The average weight of the TRIM24 overexpression group was 1.614 g, which was significantly higher than that of the control group, with an average body weight of 0.694 g (Fig. 4b and c). Compared with the tumors of control CaOV-3 cells origin, an increase in TRIM24 and Ki-67 expression were observed in the tumors derived from TRIM24 overexpressed CaOV-3 cells (Fig. 4d). These results showed that overexpression of TRIM24 promoted the proliferation of EOC cell in vivo.

### TRIM24 facilitates cell migration and induces EMT in EOC

**Fig. 5:**
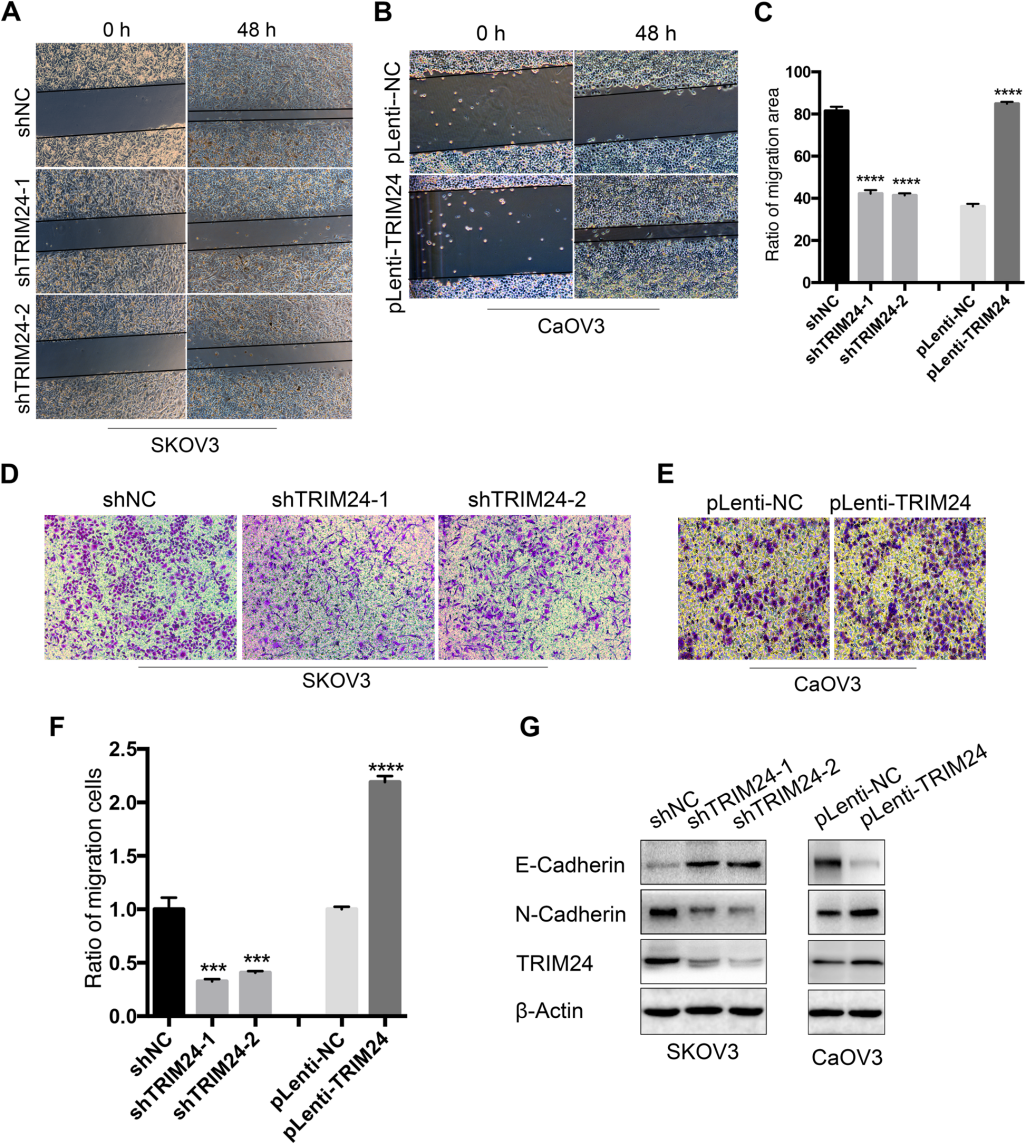
TRIM24 facilitates cell migration and induces EMT in EOC. **a** Wound-healing experiments showed that knockdown of TRIM24 expression in SKOV3 cells could inhibit cell migration. **b** Wound-healing experiments showed that overexpression of TRIM24 in CaOV-3 cells could promote cell migration. **c** The histogram of the ratio of cell migration area to scratch area of **a** and **b** (****P < 0.0001). **d** Transwell experiments showed that knockdown of TRIM24 expression in SKOV3 cells could inhibit cell migration. **e** Transwell experiments showed that overexpression of TRIM24 in CaOV-3 cells could promote cell migration. **f** Counts of the transmembrane cells in **c** and **d** and the difference was statistically significant (****P < 0.0001, ***P < 0.001). **g** Western blot showed that knockdown of TRIM24 in SKOV3 cells could promote the expression of E-cadherin and inhibit the expression of N-cadherin, while overexpression of TRIM24 in CaOV-3 cells could promote the process of EMT

To examine the effect of TRIM24 on EOC cell migration. Firstly, we carried out wound-healing experiments and the results showed that knockdown of TRIM24 expression in SKOV3 cells could inhibit cell migration, while overexpression of TRIM24 in CaOV-3 cells could promote cell migration (Fig. 5a and b). In SKOV3 control group, the cell migration area accounted for 81.43% of the scratch area, while in TRIM24 interference group, the cell migration area accounted for 42.2% and 41.4% of the scratch area, respectively. In CaOV-3 cells, the ratio of migrating cell area to scratch area in the TRIM24 overexpression group was 84.9%, while that in the control group was only 36.16% (****P < 0.0001, Fig. 5c). Further, we performed transwell chamber experiments, which also confirmed that TRIM24 has the ability to promote the migration of EOC cells (Fig. 5d and e). In CaOV-3 cells, the number of transmembrane cells in the TRIM24 overexpression group was 2.19 times higher than that in the control group, however, in SKOV3 cells, the migration ability of cells after interference of TRIM24 was significantly reduced, which was 0.326 times and 0.408 times that of the control group, respectively (****P < 0.0001, ***P < 0.001, Fig. 5f).

To determine whether TRIM24 is associated with EMT in EOC cells, we detected the key proteins (E-Cadherin and N-Cadherin) associated with EMT by Western blot. The results suggest that knocking down TRIM24 in SKOV3 cells can increase the expression of E-Cadherin and decrease the expression of N-Cadherin; conversely, overexpression of TRIM24 in CaOV-3 cells can inhibit the expression of E-Cadherin and promote the expression of N-Cadherin (Fig. 5g). These results suggested that TRIM24 might induce EMT in EOC cells.

## Discussion

Although great progress has been made in surgery and chemotherapy of EOC in recent years, due to lack of obvious symptoms and effective screening methods, early diagnosis of EOC is still difficult, local infiltration and distant metastasis of tumors are easy to occur after surgery, treatment failure and disease progression are still quite frequent, which seriously affect the quality of life and survival time of patients. Finding new and effective biomarkers associated with clinical and prognostic features of EOC are important for diagnosis and treatment.

Tripartite Motif-containing protein 24 (TRIM24), also named Transcription Intermediary Factor 1 alpha (TIF1a) [16]. Due to the heterogeneity of tissue differentiation, TRIM24 acts as a tumor suppressor or tumor promoter in different tumors. Jiang et al. demonstrated that TRIM24 suppresses development of spontaneous hepatic lipid accumulation and hepatocellular carcinoma in mice [17]. Meanwhile, several reports have associate the abnormal high expression of TRIM24 with the progression and poor survival of cancer such as breast cancer, gastric cancer, glioblastoma and clear cell renal cell carcinoma [18–21]. IACS-9571, a selective high affinity small molecule inhibitor targeting at the bromodomain domain of TRIM24, has been found and is expected to be put into further clinical research, which provides the possibility for TRIM24 to become a clinical therapeutic target of tumor [22]. Here, we try to explain the significance of TRIM24 in EOC, especially prognosis. Data from Oncomine show that the TRIM24 mRNA expression was obviously elevated in ovarian serous cystadenocarcinoma compared to normal ovary tissues. Furthermore, immunohistochemistry showed that the EOC tissues exhibited significantly higher expression level of TRIM24 protein than the normal ovarian tissues. Therefore, it is reasonable to consider that upregulated TRIM24 exhibits oncogenic function in EOC.

Going on step further to confirm the oncogenic role of TRIM24 in EOC, the relationship between TRIM24 expression and corresponding clinicopathological parameters was determined and our results revealed that elevated TRIM24 expression was closely correlated with serum CA-125, metastasis, FIGO stage and Ki-67 level. Kaplan–Meier survival analysis showed that TRIM24 expression increased inversely with the clinical prognosis of patients with EOC, which was consistent with previous study in that the higher level of TRIM24 is correlates with tumor metastasis and worse survival in EOC patients [23]. Since the clinicopathological parameters noted above represent the deterioration and development of the tumor, it offers more evidence that TRIM24 is a factor related to EOC progression.

To further confirm the function of TRIM24 in EOC, we investigated the relationship between TRIM24 and the biological behavior of EOC cell lines. In SKOV3 cell line with high expression of TRIM24, we silenced TRIM24 expression by shRNA, while in CaOV-3 cell line with low expression of TRIM24, we artificially overexpressed TRIM24. CCK-8 and colony formation assays showed that TRIM24 overexpression promoted EOC cell growth and colony formation, while TRIM24 knockdown inhibited EOC cell growth and colony formation. In addition, tumorigenic experiments in nude mice confirmed the discovery of proliferation experiments in vitro, and showed that TRIM24 knockdown inhibited tumor growth in vivo. These results are consistent with previous studies about TRIM24 in other cancers. As a new E3 ligase, TRIM24 ubiquitinates p53 to promote its degradation [7]. Loss of TRIM24 in human breast cancer cells leads to p53 dependent apoptosis [6]. High expression of TRIM24 is associated with poor survival in breast cancer patients. TRIM24 can also act as a synergistic activator of estrogen receptor (ER) to promote the proliferation of breast cancer cells, thereby promoting the occurrence of breast cancer [8]. By reprogramming of glucose metabolism, exogenous expression of TRIM24 dramatically accelerated cellular proliferation and induced malignant transformation in immortalized human mammary epithelial cell [21]. Yu et al. reported that TRIM24 promoted the survival of clear cell renal cell carcinoma cells both in vivo and in vitro [20]. We also demonstrated that TRIM24 facilitates cell migration and induces EMT in EOC. Studies in hepatocellular carcinoma and in non-small cell lung cancer cell lines had shown that down-regulation of TRIM24 induced by small interfering RNA can reduce the ability of migration and invasion, and reduce the expression of EMT-related proteins [24, 25]. These results suggest that TRIM24 plays an important role in the proliferation and metastasis of EOC cells.

Moreover, recent studies on various tumor models have shown that TRIM24 may promote tumorigenesis by affecting Akt pathway and cell cycle. Silencing TRIM24 expression in cervical cancer can inhibit the epithelial mesenchymal transition by regulating NF -κB expression and Akt pathways [26]. In gastric cancer cells, TRIM24 promotes cell growth and induces chemotherapy resistance in an Akt dependent manner [27]. Similarly, TRIM24 is involved in the regulation of Akt signaling pathway in glioblastoma, TRIM24 directly activate the expression of PIK3CA gene and then enhance phosphatidylinositosine3-kinase (PI3K)/Akt signal transduction [15]. Our study showed that overexpression of TRIM24 increased the level of p-Akt in EOC cells, while interfering TRIM24 reduced the level of p-Akt, suggesting that TRIM24 may promote the tumor progression of EOC by promoting phosphorylation of Akt.

## Conclusions

Our study identified that TRIM24 expression is increased in EOC, and it is associated with undesirable clinicopathological features and worse prognosis of patients. Mechanism explore found that TRIM24 may promote the proliferation and metastasis of EOC cells through regulating the level of p-Akt and facilitating epithelial mesenchymal transition. Our research enriches the molecular mechanism of EOC development, and provides new diagnosis and treatment strategies for EOC.

## Materials and methods

### Patients and specimens

All patients were enrolled with written informed consents and the study was reviewed and approved by the Ethics Committee of Obstetrics and Gynecology Hospital of Fudan University. Paraffin-embedded samples of 230 cases of primary EOC lesions and 39 cases of metastatic lesions were obtained from 230 patients who diagnosed with EOC and underwent surgery in the Obstetrics and Gynecology Hospital of Fudan University between 2010 and 2015. Primary lesions were defined as tumor samples from the fallopian tube or ovary; metastatic lesions were defined as tumor samples derived from the peritoneum or greater omentum. Another 38 specimens from normal ovarian tissues were obtained as control. Fresh tissue samples for qRT-PCR and Western blot experiments were collected and stored in liquid nitrogen. The histological diagnosis of the samples were determined by two qualified pathologists in a double-blind manner and classified according to WHO classification of ovarian tumors (2020). Clinical and histopathological data including patient age, FIGO stage, histology type, tumor diameter, serum CA-125, ascitic volume, metastasis status, Ki-67 and p53 immunohistochemical (IHC) score were extracted from medical records. No patients received any preoperative anti-cancer chemotherapy. Each patient was followed until March 2020, with the longest follow-up up to 100 months. The tissue microarrays (TMAs) that include all the above samples were prepared and utilized in this study. TMAs were manufactured by Servicebio (Wuhan, China).

### Immunohistochemistry and evaluation

The TMAs were deparaffinized and rehydrated by using xylene and a graded series of ethanol. Antigen repair was performed in citric acid buffer (PH6.0). Then, the slices were incubated at room temperature in 3% (v/v) hydrogen peroxide solution to block the activity of endoperoxidase. Nonspecific sites were blocked up with 3% bovine serum albumin (BSA), and incubated with a primary rabbit monoclonal anti-TRIM24 antibody (1:200 dilution, ab174287, Abcam, England). The sections were then incubated with horseradish peroxidase-conjugated anti-rabbit secondary antibody (Dako, America). Colors were presented with a DAB kit (Dako, America). Then, nucleus was counterstained with hematoxylin, dehydration and mounting. TBS containing 1% BSA was used as a substitute for the primary antibody as a negative control.

TRIM24 immunohistochemical staining was scored according to intensity and percentage of TRIM24-positive cells by two experienced pathologists independently. Staining intensity was graded from 0 to 2(0, negative; 1, weak; 2, strong). The percentage of TRIM24-positive cells was also scored from 1 to 4 (0–10%, 11–25%, 26–75%, 76–100%). A score ranging from 0 to 8 was calculated by multiplying the staining intensity score with the staining percentage of TRIM24-positive cells score, resulting in a low (0–3) level or a high (4–8) level for each sample.

### Cell lines and cell culture

The human EOC cell lines A2780, HO-8910, CaOV-3, and SKOV3 were obtained from Cell Bank of Type Culture Collection (Chinese Academy of Sciences, Shanghai, China). The former three were maintained in RPMI 1640 (Gibico, America) and SKOV3 was maintained in McCoy 5A medium (Gibico, America), supplemented with 10% fetal bovine serum (FBS, Gibco, America) and 1% Penicillin–Streptomycin (Gibico, America) at 37℃ and 5% CO2. Human ovarian immortalized nontumorigenic human ovarian surface epithelial (IOSE) were originally obtained from the ATCC.

### Cell transfection

Lentiviral vector of pLenti-CMV-3FLAG-PGK-Puro was purchased from Shanghai Obio Technology Company, and the cDNA of TRIM24 was presented by Han Jiahuai laboratory. Lentiviral vectors pGIPZ with shTRIM24-1 (Sense Sequence: 5’- AGGACCTGTTACTATGACT-3’) and shTRIM24-2 (Sense Sequence 5’-CTTGTTAAGTTAA CACCTA -3’) were ordered from WZ Biosciences Inc. According to the manufacturer’s instruction, plasmids were transfected into cells by using Lipofectamine® 2000 transfection reagent (Invitrogen, America) in serum-free Opti-MEM (Gibico, America). Cells were screened with puromycin.

### Total RNA extraction and qRT-PCR

TRI Reagent® RNA Isolation Reagent (Sigma, T9424) was used for extracting total RNA from cultured cells following the manufacturer’s instructions. Complementary DNA (cDNA) was synthesized using the TAKARA PrimeScriptTM RT reagent Kit (TAKARA, RR047A). qRT-PCR was performed using SYBR® Select Master Mix (Applied Biosystems). The primers sequences were designed as follows: TRIM24, forward 5′-CAGCCACAAATGCCTAAGCAG-3′, reverse 5′-GTGTTGGGAACTTG GATAACTGG -3′; and glyceraldehyde 3-phosphate dehydrogenase(GAPDH): forward 5’- CTGGGCTACACTGAGCACC-3’, 5’- reverse AAGTGGTCGTTGAGGGCAATG -3’. The relative mRNA expression levels of TRIM24 were normalized to GAPDH and calculated by 2^−∆∆CT^ methods.

### Western blot analysis

Cells were lysed with RIPA Lysis Buffer, total proteins were quantified by using Pierce™ BCA Protein Assay Kit (ThermoFisher, America). According to the manufacturer’s instruction, 30 μg protein samples were separated by 10% SDS–polyacrylamide gel electrophoresis and transferred to polyvinylidene fluoride (PVDF) membranes (Millipore, America), the membranes were incubated with primary TRIM24 antibody (1:1000 dilution, ab174287, Abcam, England), E-Cadherin antibody (1:1000 dilution, ab231303, Abcam), N-Cadherin antibody (1:1000 dilution, ab76011, Abcam), phospho-Akt (Ser473) (1:2000 dilution, #4060, Cell Signaling Technology), Akt antibody (1:1000 dilution, #9272, Cell Signaling Technology) and β-actin antibody (1:2000 dilution Sigma-Aldrich) overnight at 4 °C, and then incubated with horseradish peroxidase-coupled goat-anti-mouse/rabbit IgG antibody at room temperature for 2 h. Later the bound proteins were visualized by using ECL (Millipore, America) and detected by using a ChemiDoc™ XRS + System (BIO-RAD, America).

### Cell viability assays

Each group of cells was seeded into 96-well plates at a density of 2000 cells per well. After culturing for 0, 24, 48, 72, 96 and 120 h, respectively. 10 μL of CCK8 (Dojindo, Japan) solution was added to each testing well and incubated for an additional 2 h at 37 °C. The absorbance at 450 nm was gauged using the Microplate Reader (Synergy 2; Bio Tek). The average value of the 5 testing wells was considered to be the final result of each sample.

### Colony formation assays

The TRIM24 stably overexpressing or interfering cell lines were plated into 6-wells plates (500 cells per well), and cultured for 2 weeks. Cells were then fixed with methanol and stained with 0.1% crystal violet. Colonies with more than 50 cells were counted under the fluorescence microscope.

### Tumorigenicity assays in nude mice

In each group, Five 5-week-old female BALB/c nude mice were subcutaneously injected with 2 × 10^6^ cells in 0.1 mL of PBS in the right armpit region. Five weeks after the injection, the mice were sacrificed and the weights of the tumors were measured. After the subcutaneous tumor nodules appeared in mice, the meridian of tumor was measured every week. L represents the longest diameter of tumor mass; W stands for the longest transverse diameter perpendicular to the longest diameter; the tumor volume was calculated as v = π/6 × L × W^2^.

### Wound-healing assay and cell migration

Cells were seeded in 6-well plates, until the cells growth to reach more than 90% of the confluence. A pipette tip was used to make the scratches. At the appropriate time (0, 48 h), the healing of scratches was observed and photographed under a microscope.

Transwell chambers (Corning, 3422) were used to measure cell migration ability. Cells (1 × 10^5^) were plated in the upper chambers and cultured with 100 μl serum-free medium while 600 μl of 10% serum-containing medium was added to the lower chamber. After incubation for 72 h, the migrated cells were fixed and stained with Giemsa; nine random fields were photographed with inverted microscope and the migrated cells were counted.

### Statistical analysis

All experiments were conducted in at least three copies. Data processing is done using the SPSS 19 statistical package (IBM, USA). Normally distributed quantitative data were represented as mean ± standard error of mean (SEM). Chi square test was used to analyze the correlation of TRIM24 expression and the clinicopathological features of EOC. The Kaplan–Meier method was used to assess the correlation between TRIM24 expression and patients’ outcomes. For all of the tests, P < 0.05 was considered as statistically significant.

## Data Availability

All data generated or analyzed in this study are included in this article and can be obtained from the corresponding author according to reasonable requirements.

## Abbreviations

TRIM24: Tripartite Motif-Containing 24
EOC: Epithelial ovarian cancer
IHC: Immunohistochemistry
OS: Overall survival
PFS: Progression-free survival
FIGO: International Federation of Gynecology and Obstetrics
qRT-PCR: Quantitative reverse transcription polymerase chain reaction
IOSE: Human immortalized ovarian surface epithelial
EMT: Epithelial mesenchymal transformation
